# Overexpression of FNDC4 Constrains Hepatocellular Carcinoma Progression by Promoting Cell Apoptosis and Inhibiting Cell Growth

**DOI:** 10.7150/jca.88964

**Published:** 2023-10-16

**Authors:** Ningxin Li, Huan Li, Shufen Zhou, Qingyu Zhang, Guangtao Li, Huanfa Yi, Yahui Liu, Zhanchuan Ma

**Affiliations:** 1 Central Laboratory, Lequn Branch, The First Hospital of Jilin University, Changchun, Jilin, China.; 2 Department of Hepatobiliary and Pancreatic Surgery, The First Hospital of Jilin University, Changchun, 130000, China.

**Keywords:** FNDC4, hepatocellular carcinoma, tumor suppressor, prognosis evaluation, bioinformatics analysis.

## Abstract

Hepatocellular carcinoma is one of the most common malignant tumors in the world. It has been reported that fibronection type III domain containing family plays an important role in the formation and development of a variety of tumors, but the role of FNDC4 is still unclear. In our study, we found that FNDC4 was highly expressed in normal liver tissues but abnormally expressed at low levels in liver cancer tissues. Enhanced apoptosis and decreased proliferation were shown in the FNDC4 overexpression model in HepG2 cells. In addition, FNDC4 was negatively correlated with AFP, a tumor marker of HCC, and other cancer-related genes such as AHSA1, GDF1, GPC3 and MDK. In addition, we found that FNDC4 was associated with the abundance of several tumor-infiltrating lymphocytes and the expression of chemokines and immunostimulators, and FNDC4 was enriched in response to transforming growth factor β. These results indicated that FNDC4 plays a key role in hepatocellular carcinoma progression and might be a promising biomarker for cancer diagnosis.

## Introduction

Primary liver cancer is the sixth most commonly diagnosed cancer and the fourth leading cause of cancer death worldwide in 2018, with approximately 841,000 new cases and 782,000 deaths 1. Its incidence is increasing and is an important source of cancer-related mortality worldwide. Hepatocellular carcinoma (HCC) is the most common primary liver cancer. Especially in China, the incidence of liver cancer accounts for more than 50% of the total number in the world 1. The Five-year survival of HCC is 18%, second only to pancreatic cancer 2. Due to poor prognosis among patients with HCC, there is still a need to search for more effective diagnostic and prognostic markers.

Fibronectin type Ⅲ domain containing 4 (FNDC4), also known as FLJ22362 and FRCP1, is a member of the fibronectin type III domain-containing family. The fibronectin type III domain-containing family contains five proteins, namely, FNDC1, FNDC2, FNDC3, FNDC4 and FNDC5^3^. The FNDC4 protein consists of 219 amino acids and contains three identifiable domains, namely, the N-terminal signal peptide, fibronectin type III domain and 22-amino-acid transmembrane domain. The genomic region of FNDC4 spans 3,174 bp with 7 exons. FNDC4 and FNDC5 have human homologs that are highly conserved (>95% amino acid homology) 4. Studies on irisin revealed that FNDC5 is abnormally expressed in a variety of tumors and is of great significance for the occurrence and development of tumors, including gastrointestinal system cancers and breast and ovarian cancer 5, 6. Studies have shown that the expression level of FNDC5 in liver cancer tissue is significantly higher than that of the normal control group, and FNDC5 can promote the proliferation of HCC cells and inhibit their apoptosis 7. However, previous studies on FNDC4 have mainly focused on its functions in reducing the inflammatory response, inhibiting osteoclast formation and bone resorption, improving insulin resistance and reducing adipogenesis. However, there are still few reports on its specific role and potential mechanism in the growth and metastasis of HCC. Through bioinformatics analysis, we found that compared with that in normal liver tissue, the expression level of FNDC4 in liver cancer tissues was significantly downregulated. We verified that the expression of FNDC4 in HCC tissues was significantly reduced in patient tissues. By constructing an overexpression model of FNDC4, we found that cell proliferation was weakened and apoptosis was enhanced in HepG2 cells overexpressing with FNDC4. In addition, we also found that the expression of FNDC4 was negatively correlated with the content of AFP, which can reflect the degree of cancelation of cells. There was also a negative correlation between the expression of FNDC4 and overall survival (OS) (*p* < 0.05), while there was no statistical significance in progression-free survival (PFS) and relapse-free survival (RFS). Our results showed that FNDC4 had certain clinical research value and was expected to become a potential target or biomarker for the diagnosis and treatment of liver cancer.

## Materials and Methods

### The Human Protein Atlas

The Human Protein Atlas (http://www.proteinatlas.org/) is an effort to map the location of all human proteins, and it contains a large number of histological images of sections from human tissue. On this website, we searched with FNDC4 as a key word and obtained a series of atlas of the expression levels of FNDC4 RNA and protein in different tissues of the human body. We also obtained the images of histological sections of FNDC4 from human normal liver tissues (HPA051804: Female, age 54, id:3402; Male, age 67, id:1720) and the pathological sections of FNDC4 from liver cancer tissues of different patients (HPA051804: Female, age73, id:2279; Male, age 59, id:3625).

### UALCAN

UALCAN (http://ualcan.path.uab.edu) is an online tool, using the TCGA level of RNA-seq and clinical data from 31 cancer types 8, 9. In the TCGA gene analysis, we entered the gene name FNDC4 and selected liver hepatocellular carcinoma (LIHC) as the cancer name for retrieval. Then we were able to obtain the comparison image of the expression level of FNDC4 in liver cancer tissues with those in normal liver tissues based on clinicopathological characteristics such as patient's gender, weight, age, tumor grade, and individual cancer stage (http://ualcan.path.uab.edu/cgi-bin/TCGAExResultNew2.pl?genenam=FNDC4&ctype=LIHC).

### TIMER: Tumor IMmune Estimation Resource

TIMER (http://timer.cistrome.org/) is an online tool which is used to study tumor immunological, clinical and genomic features comprehensively. The Correlation module can draw the expression scatterplots between a pair of user-defined genes in a given cancer type, together with the Spearman's rho value and estimated statistical significance 10, 11. In the correlation module, we inputted a pair of target genes and selected the cancer type as LIHC, and then we were able to determine the relationship between the expression of FNDC4 with the expression of AFP and other tumor-related genes such as GPC3, MDK and DKK1.

### TISIDB

The TISIDB is a web portal for tumor and immune system interaction and by using the multidimensional profiling data for 30 cancer types from TCGA, TISIDB pre-calculated the relation between the immune features (e.g., lymphocytes, immunomodulator, chemokines) and expression, copy number, methylation, and mutation of any gene 12. In this study, we checked the “lymphocyte”, “immunomodulator”, and “chemokine” tab to evaluate the possible correlation of the expression of FNDC4 with the abundance of tumor-infiltrating lymphocytes, immunostimulators and chemokines in liver hepatocellular carcinoma (http://cis.hku.hk/TISIDB/browse.php?gene=FNDC4).

### The Kaplan-Meier Plotter

Using Kaplan-Meier Plotter database (http://www.kmplot.com), we entered the gene name on the official website to obtain FNDC4 gene expression data and survival information in HCC patients to evaluate the prognostic value of FNDC4 mRNA expression 13. We filled in FNDC4 in the gene symbol column and selected overall survival (OS), relapse-free survival (RFS), progression-free survival (PFS) and disease-free survival (DFS) in the survival column to obtain a series of Kaplan-Meier survival maps (http://kmplot.com/analysis/index.php?p=service&cancer-liver_rnaseq). We also divided patients by gender when studying each survival measure (e.g., OS, OS Female and OS Male).

### KOBAS (KEGG Orthology Based Annotation System)

KOBAS (http://kobas.cbi.pku.edu.cn/) consists of two parts called the "annotation module" and the "enrichment module", and the enrichment module gives an answer about which pathways and GO terms are statistically significantly associated with the input genes 14. In the gene-list enrichment module, we filled in FNDC4 in the gene symbol column and selected GO to obtain the GO enrichment analyses results of FNDC4.

### GeneMANIA Database

GeneMANIA (https://genemania.org/) can be used to search other genes that are related to a set of input genes, by using a large set of functional association data 15. It constructed protein-protein interaction (PPI) networks in terms of physical interaction, co-expression, predicted, colocalization, common pathway, genetic interaction, and shared protein domains. In this study, we searched FNDC4 to establish an interaction network of FNDC4 related genes and try to generate hypotheses about gene function based on the above aspects.

### Cell culture and transfection

HepG2 cells were purchased from the Shanghai Zhong Qiao Xin Zhou Biotechnology Co.,Ltd, and authenticated by short tandem repeat (STR) analysis, cells were negative for mycoplasma detection. HepG2 cells were cultured in DMEM medium contain 10% fetal bovine serum (Yeasen, China). The overexpressing plasmid of FNDC4 were purchased from the genepharma (Suzhou, China). Cells were seeded in 6-well plates for 24 hours. Then, FNDC4 overexpression plasmid or negative control plasmid were mixed with Lipofectamine 3000 reagent (Invitrogen, USA) according to the manufacturer's protocol.

### Cell viability detection assay

Transfected cells were scraped with a sterile 200 μl pipette tip, cell debris was removed, and cells were cultured in DMEM containing 0.5% fetal bovine serum. The migration area of the cells was imaged at 0 hours and 48 hours to assess the migration ability. Cells were collected at 48 hours to detect the apoptotic rate according to the manufacturer's protocol (Annexin V-FITC/PI Apoptosis Detection Kit, Yeasen, China). For the CCK-8 assay, CCK-8 buffer was added to transfected cells in 96-well plates for the indicated times. The absorbance at 450 nm was measured by a multimode reader (Synergy HT, BioTek, USA).

### mRNA and miRNA Detection

Human specimens were obtained from the Department of Biobank, Division of Clinical Research, The first hospital of Jilin University. In this study, 10 patients with hepatocellular carcinoma were recruited from the Department of Biobank, Division of Clinical Research, The First Hospital of Jilin University, between July 2020 to June 2021. The diagnosis of HCC (Hepatocellular carcinoma) was according to the consensus guidelines of the Milan criteria and based on the clinical symptoms, results of histological analysis and endoscopy, and physician's assessment 16, 17. The exclusion criteria were pregnancy, acute infection, and drug treatments. All participants were given written informed consent, and all procedures in this study were approved by the ethics committee of the First Hospital of Jilin University. And the changes in the manuscript were all highlighted for viewing purposes. Table 2 lists the clinical parameters of the HCC patients. Written informed consent was provided by all study participants. The study protocol was approved by the ethics committee of the First Hospital of Jilin University. Total RNA was extracted from the sample tissues with Trizol (Invitrogen) reagent. The cDNA was synthesized using TransScript First-Strand cDNA Synthesis SuperMix (TransGen Biotech). qRT-PCR was performed using SYBR Green Kit (TransGen Biotech) under an ABI StepOnePlus system (Applied Biosystems) to evaluate the relative expression of target genes. Relative mRNA/miRNA expression was calculated using the 2-ΔΔCT method. The sequence set of related primers (Table 1) is shown in Table 1.

### Ethics Statement

The current study has been submitted to and approved by the ethics committee of the First Hospital of Jilin University.

### Statistical Analysis

Statistical significance was determined by the unpaired, two-tailed Student's t test analysis using Prism 7.0 (GraphPad Software). And *p* < 0.05 was considered statistically significant. Data were calculated on the basis of three independent experiments unless otherwise stated. Data were expressed as mean ± SD.

### Statement

All method in this study were performed in accordance with the relevant guidelines and regulations.

## Results

### Expression Level of FNDC4 was High in Normal Liver Tissues

**Figure 1:**
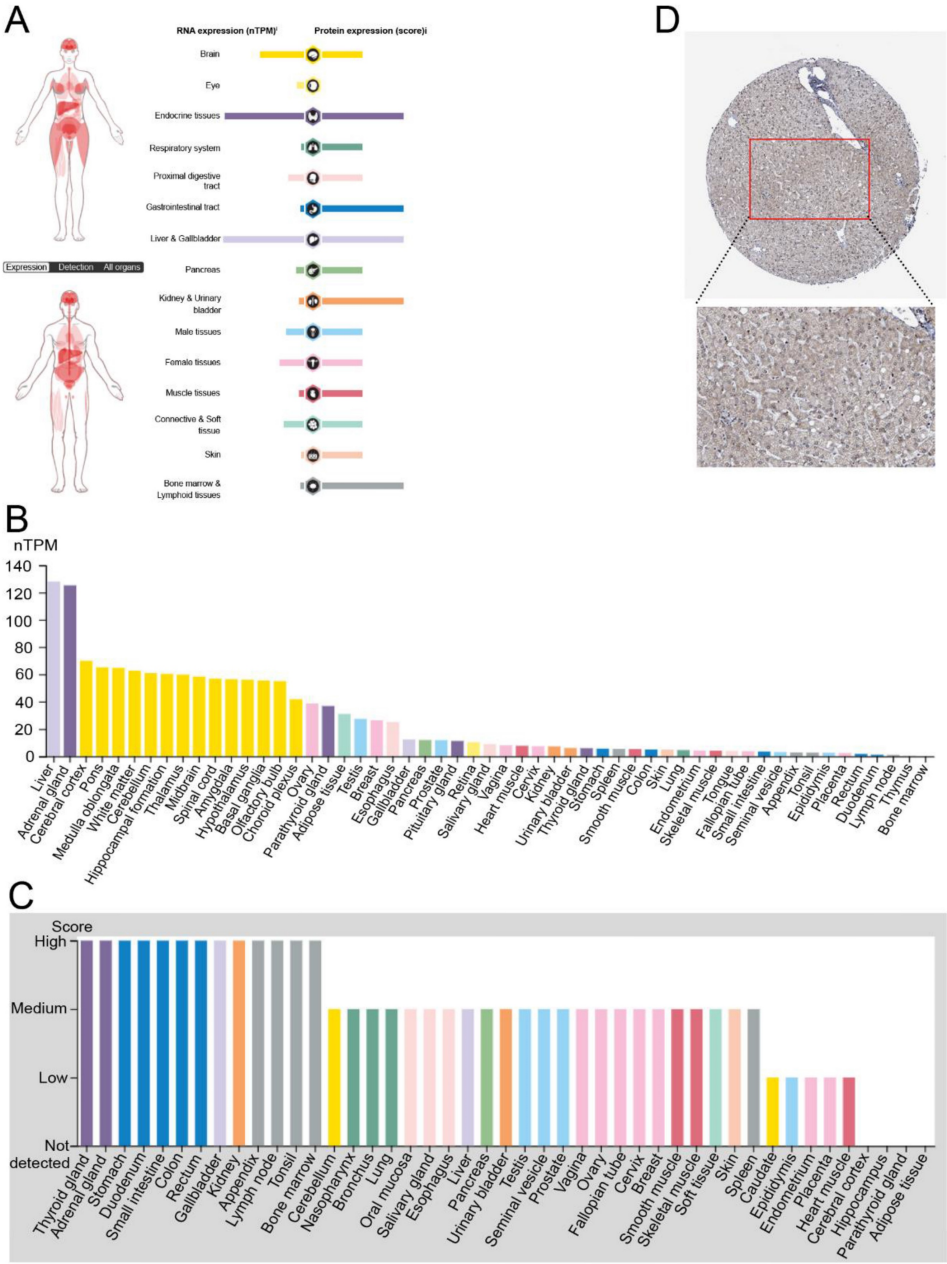
** High expression of FNDC4 in normal liver tissues. A.** The distribution of FNDC4 in human organs at the mRNA and protein level. **B** High level of FNDC4 could be detected in liver at the RNA transcriptional level. **C** FNDC4 could be detected in all tissues and organs at the protein expression level. **D** Immunohistochemical results confirm the high expression of FNDC4 protein in liver tissue. Data were extracted from The Human Protein Atlas database with the keyword “hepatocellular carcinoma”

To investigate the role of FNDC4, especially in hepatocellular carcinoma, we first analyzed its transcriptional levels in normal tissues of different organs by using The Human Protein Atlas Database (Figure 1). We found that the RNA and protein expression levels of FNDC4 were high in normal liver tissues compared with other organs (Figure 1A). In particular, the RNA transcriptional level of FNDC4 in liver tissues ranked first among all the tissue samples included (Figure 1B). Additionally, the protein expression level of FNDC4 was moderate in the contained tissues and organs (Figure 1C). In addition, the high expression of FNDC4 protein in liver tissue was also confirmed by immunohistochemical results (Figure 1D). The liver tissue sections showed a large amount of intensely stained FNDC4 protein in liver cells, indicating that high expression of FNDC4 may have some potential effects on liver cells. In summary, we found that FNDC4 was highly expressed in normal liver tissue.

### Decreased Expression of FNDC4 in Hepatocellular Carcinoma Tissues

**Figure 2:**
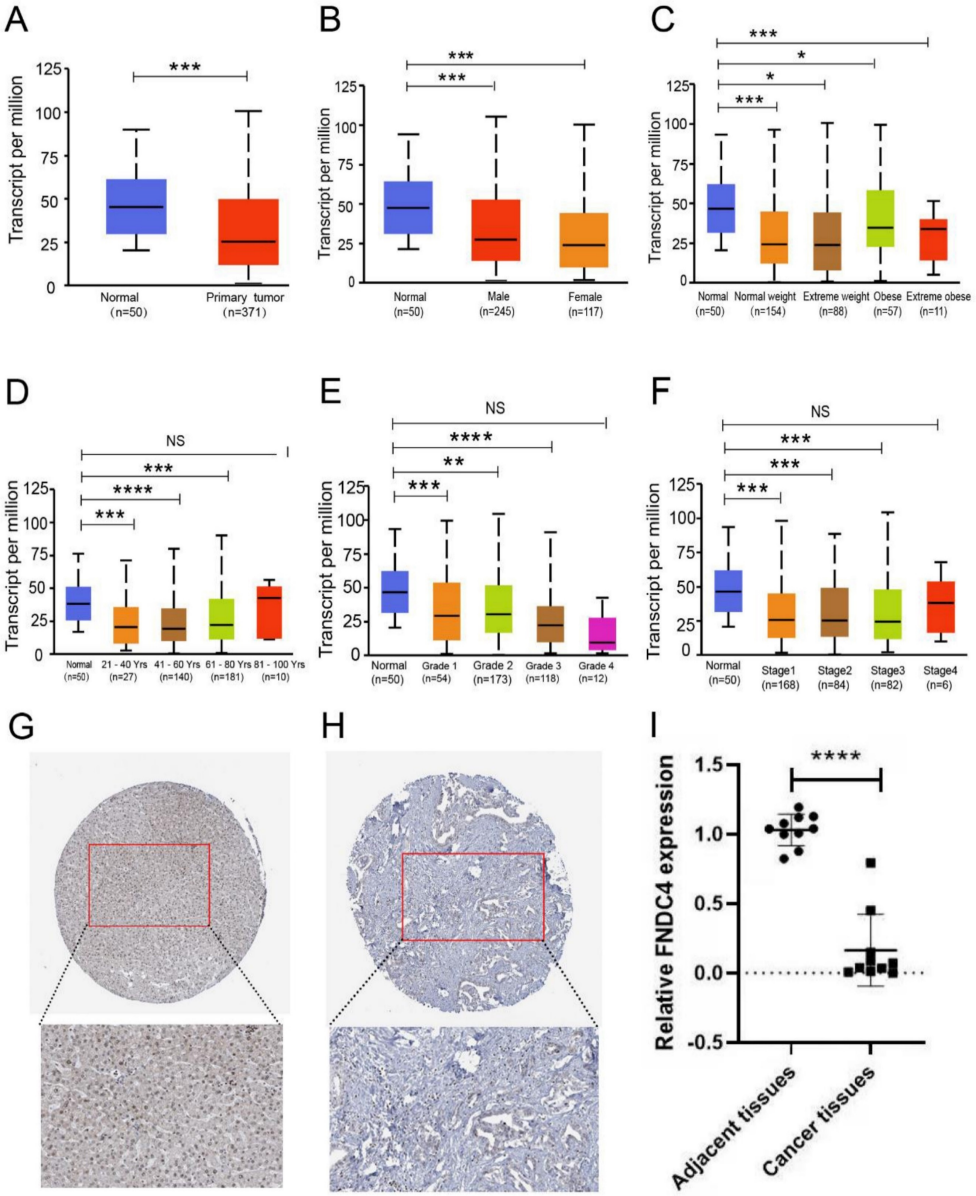
**Decreased expression of FNDC4 in liver cancer tissues. A.** Expression level of FNDC4 is significantly downregulated in liver cancer tissues at the RNA transcriptional level. *** p<0.001. **B-F.** Differential expression of FNDC4 in liver cancer tissues and normal tissues based on patient's gender (B), weight (C), age (D), tumor grade (E), and individual cancer stage (F). * *p*<0.05. ** *p*<0.01. *** *p*<0.001. NS: No statistical significance. The expression level of FNDC4 was analyzed and collected by the UALCAN database. Immunohistochemical pathological sections of FNDC4 in hepatocellular carcinoma tissue (H) show lower expression than that in normal tissues (G), data were extracted from The Human Protein Atlas database with the keyword “hepatocellular carcinoma”. I Comparison of FNDC4 mRNA expression levels in HCC tissues with those in adjacent tissues detected by using real-time qPCR. **** *p*<0.0001

To further research the correlation between FNDC4 and liver hepatocellular carcinoma, we compared the expression level of FNDC4 in liver cancer tissues with that in normal liver tissues (Figure 2A) based on patient sex (Figure 2B), weight (Figure 2C), age (Figure 2D), tumor grade (Figure 2E), and individual cancer stage (Figure 2F). We found that the transcription level of FNDC4 was significantly downregulated in hepatocellular carcinoma versus normal tissue. In The Human Protein Atlas Database, the immunohistochemical pathological sections of FNDC4 in hepatocellular carcinoma also showed that, compared with normal liver tissue, FNDC4 was expressed at a low protein level in cancer tissues (Figure 2G-H). In addition, from the clinical sample detection results, we verified that the mRNA expression level of FNDC4 was significantly reduced in HCC tissues compared with adjacent tissues (*p*<0.001, Figure 2I). Collectively, our results suggested that FNDC4 had a certain correlation with the occurrence of hepatocellular carcinoma. The expression of FNDC4 in hepatocellular carcinoma was significantly decreased. FNDC4 may act as a tumor suppressor gene in hepatocellular carcinoma.

### The expression of FNDC4 is negatively correlated with AFP, GPC3, MDK and DKK1

**Figure 3:**
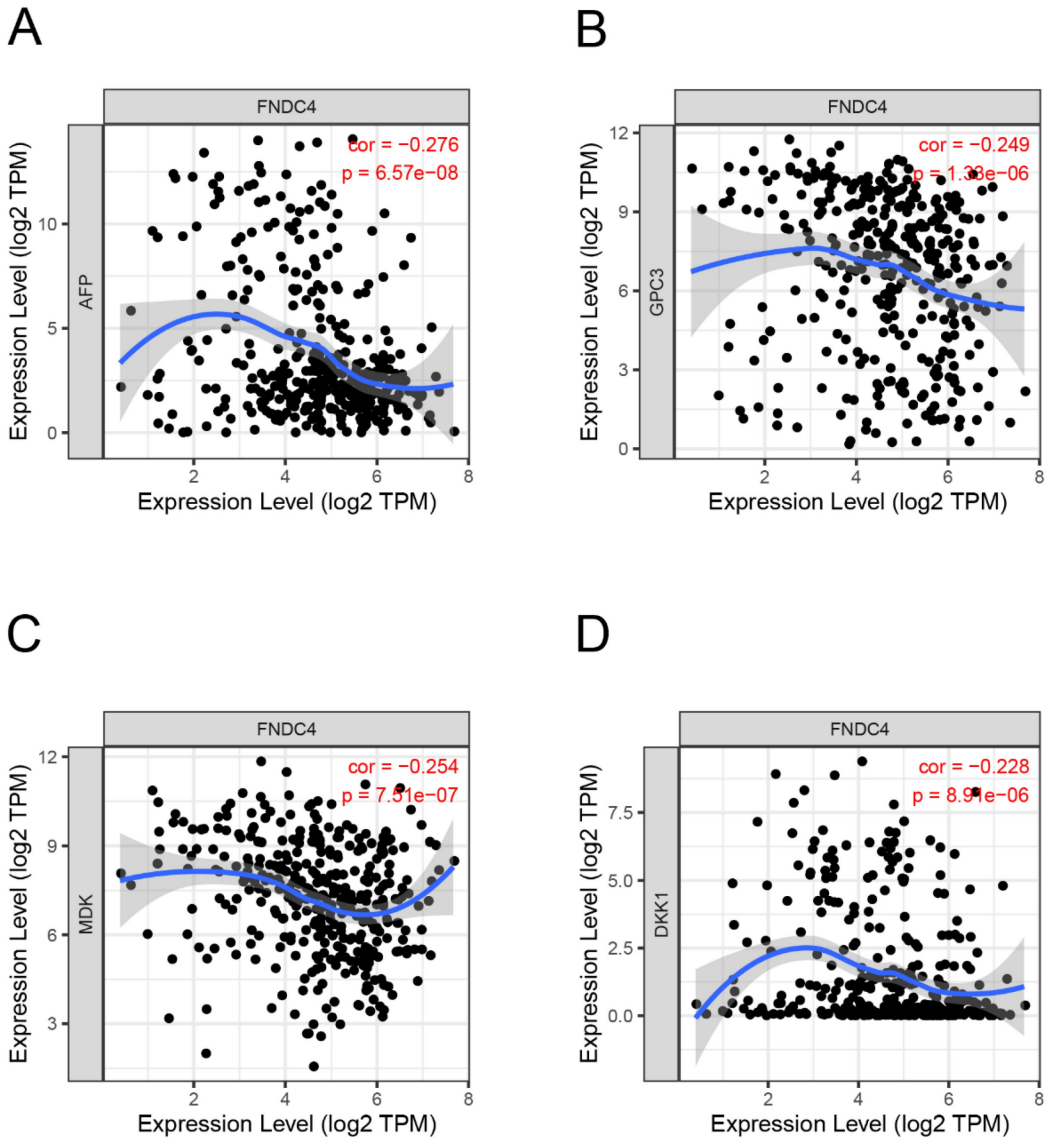
** Correlation analysis of FNDC4 and the target genes in HCC.** The expression of FNDC4 is negatively correlated with AFP (A, Spearman: r=-0.276, *p*=6.57e-8), GPC3 (B, Spearman: r=-0.249, *p*=1.33e-6), and MDK (C, Spearman: r=-0.254, *p*= 7.51E-7). and DKK1 (D, Spearman: r=-0.228, *p*= 8.91E-6). Correlation analysis was performed by the TIMER database.

To explore whether FNDC4 could act as a biomarker in HCC, we studied the correlation between the expression of FNDC4 and AFP, a primary hepatocellular carcinoma-specific tumor marker, and other liver cancer-related genes, such as Glypican 3 (GPC3), Midkine (MDK) and Dickkopf WNT signaling pathway inhibitor 1 (DKK1). By using the TIMER database, we found that the expression levels of AFP (Figure 3A), GPC3 (Figure 3B), MDK (Figure 3C) and DKK1 (Figure 3D) were negatively correlated with FNDC4. These results suggested that the expression of FNDC4 was associated with the inhibition of liver cancer. However, since the correlation between AFP, GPC3, MDK, DKK1 and FNDC4 is not high, the sensitivity of FNDC4 as a tumor marker still needs further study.

### FNDC4 might Regulate Tumor-infiltrating, Lymphocyte Abundance and the Expression of Chemokines and Immune Stimulators

**Figure 4:**
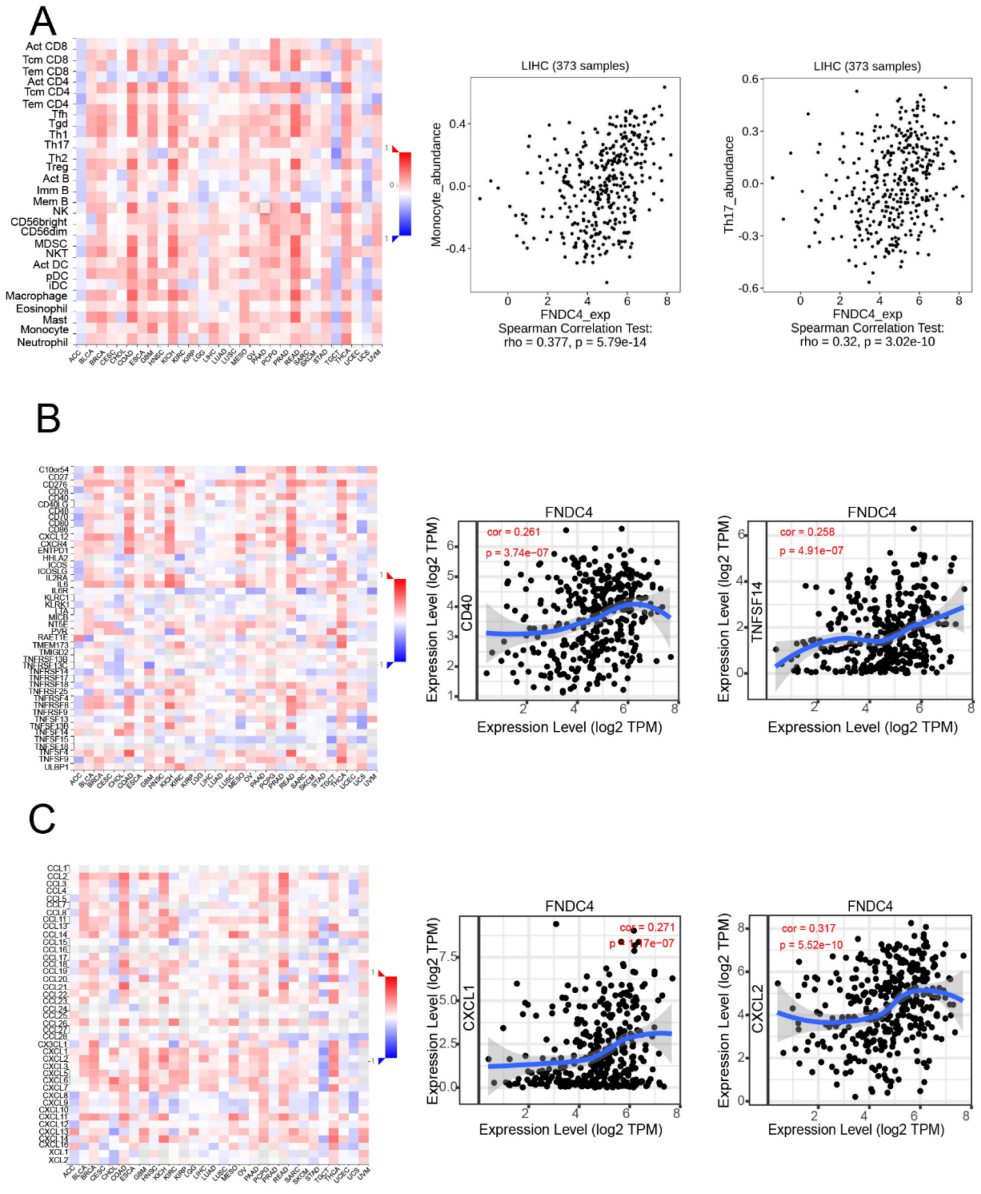
FNDC4 regulates tumor-infiltrating lymphocyte abundance and the expression of chemokines and immune stimulators. **A.** Expression of FNDC4 in HCC patients is correlated with the abundance of tumor-infiltrating lymphocytes (TILs), including monocytes (Spearman: r= 0.377,* p*= 5.79E-14) and Th17 cells (Spearman: r= 0.32, *p*= 3.02E-10). **B.** Expression of FNDC4 is correlated with the expression of CD40 (Spearman: r=0.261, *p*=3.74E-7) and TNFSF14 (Spearman: r= 0.258, *p*=4.91E-7). **C.** Expression of FNDC4 is correlated with CXCL2 (Spearman: r=0.317, *p*=5.52E-10) and CXCL1 (Spearman: r=0.271, *p*=1.17E-7). Heatmaps were analyzed by the TISIDB database.

We further investigated whether FNDC4 was a significant factor related to immune infiltration in hepatoma by using the TISIDB and TIMER databases (Figure 4). We found that FNDC4 expression was correlated with several tumor-infiltrating lymphocytes (TILs) in hepatoma patients, including monocytes (Spearman: r=0.377, *p*=5.79e-14) and Th17 cells (Spearman: r=0.32, *p*=3.02e-10) (Figure 4A). We also explored the correlation between FNDC4 expression and immunostimulators in HCC, such as CD40(Spearman: r=0.261, *p*=3.74E-7) and TNFSF14 (Spearman: r=0.258, *p*=4.91E-7). In addition, FNDC4 expression was also associated with several kinds of chemokines, including CXCL2 (Spearman: r=0.317, *p*=5.52e-10) and CXCL1 (Spearman: r=0.271, *p*=1.17e-7) (Figure 4C). These findings explained the inhibitory effect of FNDC4 on HCC and its effect on the prognosis of HCC patients.

### High expression of FNDC4 was associated with poor prognosis in HCC patients

**Figure 5:**
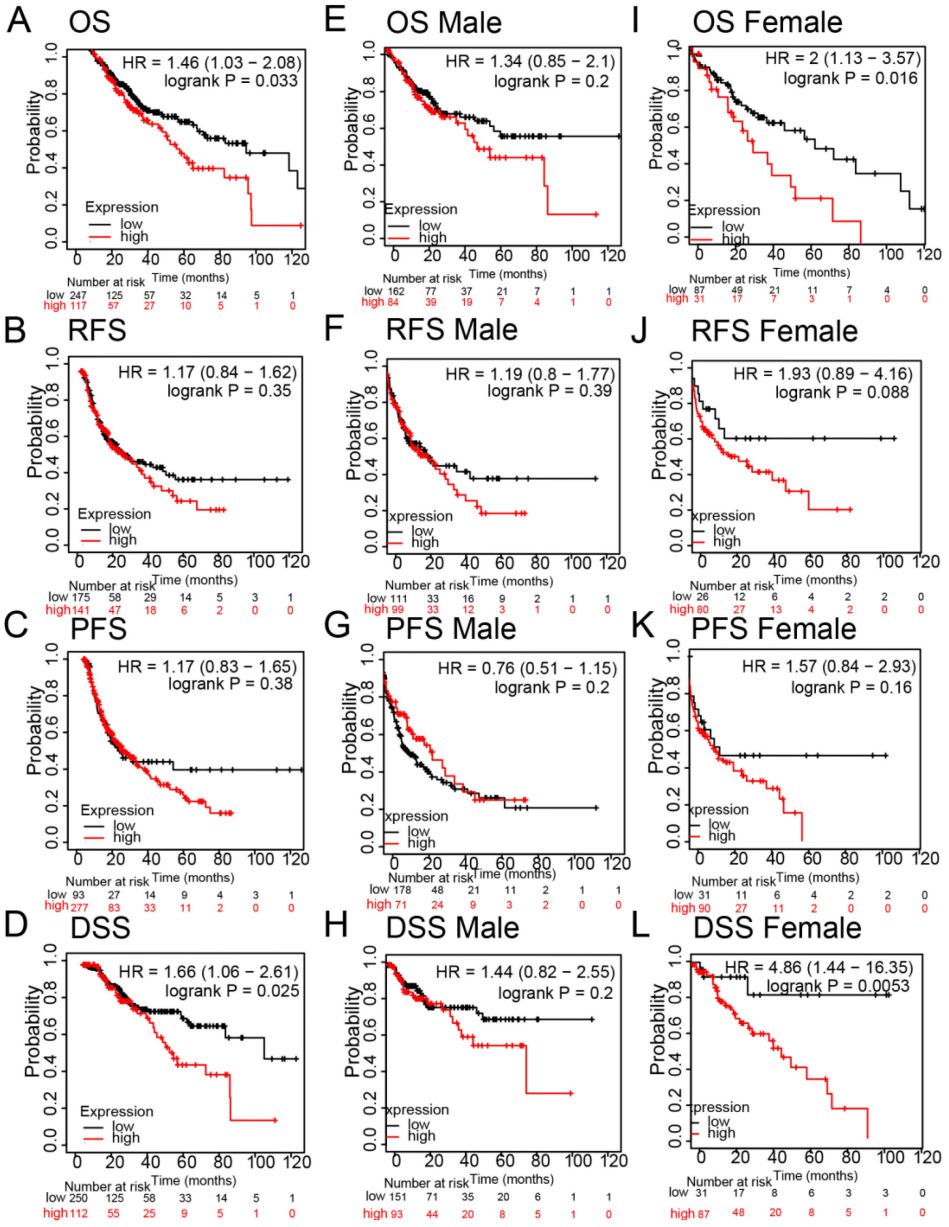
** High expression of FNDC4 is associated with poor prognosis in HCC patients.** Kaplan-Meier plotter indicated the roles of FNDC4 on OS (A, E, I), RFS (B, F, J), PFS (C, G, K), and DDS (D, H, L) in patients with lung cancer. Data were analyzed through the KM plotter database. OS: overall survival; PFS: progression-free survival; RFS: relapse-free survival; DDS: disease-specific survival.

Regarding HCC survival, we examined the influence of FNDC4 on overall survival (OS), progression-free survival (PFS), relapse-free survival (RFS) and disease-specific survival (DSS) for the prognostic analyses of hepatocellular carcinoma using the Kaplan-Meier Plotter database. The results showed that high expression of FNDC4 was negatively correlated with OS (Figure 5A), while there was no statistical significance in RFS (Figure 5B, Figure 5F, Figure 5J) and PFS (Figure 5C, Figure 5G, Figure 5K). Notably, high expression of FNDC4 predicted a poor DSS with HCC patients (Figure 5D), especially with female HCC patients (Figure 5L), other than male HCC patients (Figure 5H). We also found that the expression of FNDC4 was related to OS in female HCC patients (Figure 5I), but not male patients (Figure 5E). These results indicated that FNDC4 might influence patients' survival through the involvement of gonadal hormone.

### Enhanced apoptosis and decreased proliferation in HepG2 cells overexpressing FNDC4

**Figure 6:**
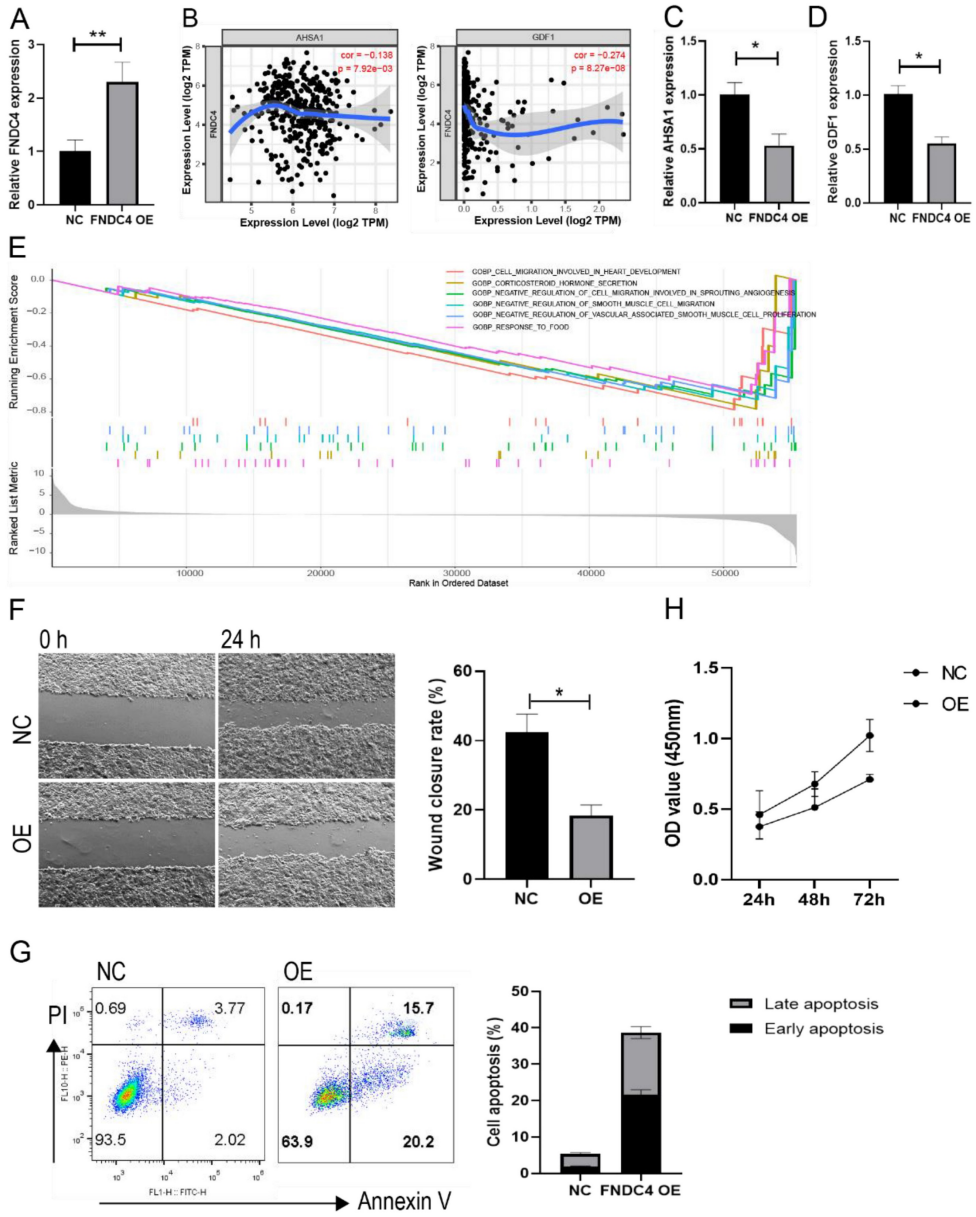
** FNDC4 overexpression led to enhanced apoptosis and decreased proliferation in HepG2 cells.** HepG2 cells were transfected with the FNDC4 overexpression plasmid or negative control (NC) plasmid, cells were cultured for 48 hours and collected to detect the expression of FNDC4 (A), AHSA1 (C), and GDF1 (D). (B) Correlation analysis of FNDC4 and AHSA1/GDF1 in the cancer tissue of LIHC patients. (E) Top significant KEGG pathways enriched by FNDC4 in GSEA. (F) Images of HepG2 cells at 0 hours and 48 hours after transfection. The apoptotic rate (G) and cell viability (H) were detected at the indicated times. * *p*<0.05. ** *p*<0.01.

Because of the decreased expression of FNDC4 in liver cancer tissues, we further constructed an overexpression model of FNDC4 in HepG2 cells (Figure 6A). We found that FNDC4 was inversely correlated with AHSA1 and GDF1 (Figure 6B), two novelly identified genes that were shown to be positively associated with tumor progression in HCC. Moreover, we confirmed that AHSA1 and GDF1 expression was decreased in HepG2 cells overexpressing FNDC4 (Figure 6C-D). In addition, enrichment analysis from TCGA liver cancer database showed that the expression of FNDC4 was associated with cell migration ability (Figure 6E). Therefore, we conducted a scratch experiment to verify the changes in the migration ability of liver cancer cells after high expression of FNDC4 and found that the wound closure rate was reduced in the overexpression model (Figure 6F). In addition, the results also indicated enhanced apoptosis ratio (Figure 6G) and decreased proliferation ability (Figure 6H) in HepG2 cells overexpressing FNDC4, which suggested that the decreased expression of FNDC4 may have a favorable effect on tumor growth.

### GO Enrichment Analysis of FNDC4-Related Biological Processes

To further study the potential molecular mechanism of FNDC4 action, the KOBAS database was used for GO enrichment analysis of the FNDC4 gene. GO analysis showed that the main biological processes of FNDC4 enrichment included the response to transforming growth factor β (GO:0071559) and the negative regulation of the inflammatory response (GO:0050728). Enriched cell components included endoplasmic reticulum (GO:0005783) and extracellular region (GO:0005615) (Table 3). We also showed the interaction network of FNDC4-related genes (Supplementary Figure 1). RNA polymerase II subunit G (POLR2G) might colocalize and interact with FNDC4. Regucalcin (RGN) might also have genetic interactions and co-expression relationships with FNDC4. These results suggest that FNDC4 might be widely involved in the development of HCC through interaction with multiple genes, highlighted the important role of FNDC4 in HCC.

## Discussion

Liver cancer is a malignant tumor with a high incidence in the human population, and hepatocellular carcinoma (HCC) accounts for approximately 80% of cases 18. Previous studies on FNDC4 have mainly focused on its functions in reducing the inflammatory response and inhibiting osteoclast formation and bone resorption, while there are still few reports on its potential function in the growth and metastasis of cancer cells. In our study, we found that FNDC4 was highly expressed in normal liver tissues but significantly downregulated in HCC tissues both in the mRNA and protein level. We also found a negative correlation between the expression levels of AFP and FNDC4, although the correlation was not high, and the expression of FNDC4 was negatively correlated with other liver cancer-related genes, such as Glypican 3 (GPC3), Midkine (MDK) and Dickkopf WNT signaling pathway inhibitor 1 (DKK1). Glypican-3 (GPC3) is a heparin sulfate proteoglycan. Studies have shown that the expression level of GPC3 is elevated in HCC patients 19, and the heparin sulfate chain of GPC3 interacts with heparin-binding growth factors and other growth factors, such as HGF and VEGF, to promote the development of HCC 20. Midkine (MDK) is a heparin-binding growth factor that plays a key role in cell growth, migration, angiogenesis and carcinogenesis 21. MDK levels in HCC patients were found to be higher than those in cirrhotic patients or healthy controls 22. The sensitivity of MDK in differentiating early HCC from cirrhosis was 90%, significantly higher than AFP's sensitivity 40% 23. Studies have found that serum DKK1 can be used as a supplement to AFP measurement in HCC diagnosis, improving the diagnostic accuracy of AFP-negative HCC patients 24. These results suggested that the expression of FNDC4 was associated with the inhibition of liver cancer.

In our study, we found that FNDC4 expression was positively correlated with the infiltration of monocytes and Th17 cells, as well as several immunostimulators, such as CD40 and TNFSF14, and some proinflammatory cytokines such as CXCL2 and CXCL1. Some previous studies confirmed that overexpression of CXCL2 inhibits cell proliferation and promotes apoptosis in hepatocellular carcinoma as CXCL2 expression was stably down-regulated in HCC patients, and its overexpression made cell cycle stagnated at G0/G1 phase, profoundly attenuated HCC cell proliferation and growth and induced apoptosis in vitro 25. High expression of CXCL2/10/12/14 indicates favorable outcomes in HCC patients and CXCL2/14 were both stably down-regulated in HCC specimens compared with adjacent normal tissues and its overexpression induced apoptosis, and played significant roles in inhibiting the proliferation, migration, and invasion of HCC cells 26. In addition, previous studies have shown that both CD40 and TNFSF14 are important tumor suppressor factors and CD40/CD40L interaction plays a critical role in the maturation of DCs in vivo, which greatly affect APC function, namely IL-12 production, in antitumor responses 27. TNFSF14, mainly expressed on activated T cells, is activated in Natural Killer (NK) cells, and immature dendritic cells (DC) 28. Yanan Ma found that TNFSF14 plays a role as a proapoptotic gene, and patients with high TNFSF14 expression in HCC showed less portal invasion and longer survival and disease-free time than those with low TNFSF14 expression 29. Therefore, we speculated that FNDC4 might play an inhibitory role in hepatocellular carcinoma by affecting the expression activity of some inflammatory cytokines, such as CD40, TNFSF14 or CXCL2. However, the specific mechanism of action remains to be further studied.

Studies have shown that transforming growth factor β (TGF-β) has a two-sided effect on tumors. In the early stage of tumor occurrence, TGF-β inhibits the oncogene c-Myc by increasing the expression of the cyclin-dependent kinase inhibitors p15 and p21 30, 31. In the advanced stage of tumors, TGF-β can create a more favorable environment for tumor invasion and metastasis 32, 33. Studies of chemically induced liver fibrosis indicate that TGF-β1 promotes the differentiation of mesenchymal cells into hepatic stellate cells and myofibroblasts to promote fibrosis 34, 35. Fibrosis is a risk factor for HCC. In our study, we found that FNDC4 is enriched in the response to TGF-β. We speculated that the stimulating effect of FNDC4 on proinflammatory cytokines and immune cells in the late liver cancer stage may aggravate the process of liver fibrosis, just as TGF-β promotes the differentiation of mesenchymal cells into hepatic stellate cells and myofibroblasts. It was reported that FNDC family of genes could be expressed in several tumor types, like breast cancer (FNDC1, FNDC8), brain and Central Nervous System cancer (FNDC3A), cervical cancer (FNDC3B), liver cancer (FNDC4), gastric cancer (FNDC5), and melanoma (FNDC6) 36, 37, which were correlated with survival of cancer patients, thus, could potentially be used as new prognostic biomarkers or promising targets for tumor therapy.

In addition, in our study, we further confirmed that overexpression of FNDC4 can enhance apoptosis and reduce proliferation of HepG2 cells in vitro. Moreover, it has been proven that the activator of HSP90 ATPase activity 1 (AHSA1) and growth differentiation factor 1 (GDF1) genes are closely related to the tumor progression of HCC. AHSA1 is a main activator of Hsp90ATPase, which is involved in the metabolism and development of tumor cells 38. A previous study showed that AHSA1was abnormally high expressed in LIHC and the knockdown of AHSA1 can decrease the proliferation, migration and invasion of HCC cells 39. This study also reported that the expression of AHSA1 was significantly positively related to the infiltration level of various immune cells, such as B cells, CD4+ T cells, CD8+T cells and Myeloid dendritic cells, greatly affecting the microenvironment in hepatocellular carcinoma 39. Similarly, GDF1, which belongs to the transforming growth factor-β, has been confirmed to be highly expressed in high-grade poorly differentiated HCCs, and closely related to poor tumor differentiation, inducing tumor lineage plasticity, and induced tumour metastasis 40. Meanwhile, in our study, we found that FNDC4 was negatively correlated with AHSA1 and GDF1, and the expression of AHSA1 and GDF1 was downregulated in HepG2 cells overexpressing FNDC4. This further confirms the hypothesis that FNDC4 acts as a tumor suppressor gene in hepatocellular carcinoma. Our study suggests that FNDC4 is a potential target or biomarker for the future diagnosis and treatment of liver cancer, providing theoretical support for subsequent relevant research, but still more data are needed.

## Conclusion

These results indicated that FNDC4 plays an inhibitory role in the progression of hepatocellular carcinoma and might be a promising diagnostic and therapeutic target for liver cancer.

## Supplementary Material

Supplementary figure.

## Figures and Tables

**Table 1:** Primer details of target genes

Primer name	Primer sequence
β-actin	
Forward Primer	5' CATGTACGTTGCTATCCAGGC 3'
Reverse Primer	5' CTCCTTAATGTCACGCACGAT 3'
FNDC4	
Forward Primer	5' CACTTCCGAACTCTCAAGGGT 3'
Reverse Primer	5' GCAGAACAGCCCAATTACAGC 3'

**Table 2:** Clinicopathological characteristics of the HCC patients

Characteristic	Number (%)
**Gender**	
Male	6 (60%)
Female	4 (40%)
**Age**	
≥60	8 (80%)
<60	2 (20%)
**Tumor size (cm)**	
≥5	6 (60%)
<5	4 (40%)
**Pathological differentiation**	
Well/moderate	3 (30%)
Poor	7 (70%)
**Lymph node metastasis**	
N0	2 (20%)
N1-3	8 (80%)
**Distant metastasis**	
M0	8 (80%)
M1	2 (20%)
**TNM stage**	
I-II	4 (40%)
III-IV	6 (60%)

TNM: tumor-node-metastasis; cm: centimeters.

**Table 3:** GO enrichment analysis of FNDC4 related biological processes.

Term	Database	ID	Total	P-value	Corrected P-value
Response to transforming growth factor beta	Gene Ontology	GO:0071559	9	2.55E-04	1.78E-03
Negative regulation of inflammatory response	Gene Ontology	GO:0050728	95	2.45E-03	8.56E-03
Endoplasmic reticulum	Gene Ontology	GO:0005783	1018	2.60E-02	6.06E-02
Extracellular space	Gene Ontology	GO:0005615	1572	4.01E-02	6.58E-02
Extracellular region	Gene Ontology	GO:0005576	1843	4.70E-02	6.58E-02
Integral component of membrane	Gene Ontology	GO:0016021	3643	9.29E-02	1.08E-01
Plasma membrane	Gene Ontology	GO:0005886	4619	1.18E-01	1.18E-01

GO enrichment analysis of FNDC4 by using KOBAS database showed that the main biological processes include the response to transforming growth factor β (GO:0071559) and negative regulation of inflammatory response (GO:0050728). The enriched cell components include endoplasmic reticulum (GO:0005783), extracellular region (GO:0005615), etc.

## Abbreviations

AHSA1: HSP90 ATPase activity 1
FNDC4: Fibronectin type Ⅲ domain containing 4
GDF1: growth differentiation factor 1
HCC: Hepatocellular carcinoma
OS: overall survival
PFS: progression-free survival
RFS: relapse-free survival
RGN: Regucalcin
TGF-β: transforming growth factor β
