# Supplementary material for: Overexpression of FNDC4 Constrains Hepatocellular Carcinoma Progression by Promoting Cell Apoptosis and Inhibiting Cell Growth
Source: J Cancer. 2023 Oct 16;14(18):3416–28. doi: 10.7150/jca.88964 (PMC10647187; doi:10.7150/jca.88964)
Supplement: Supplementary file 1 — Supplementary figure. [file jcav14p3416s1.pdf]

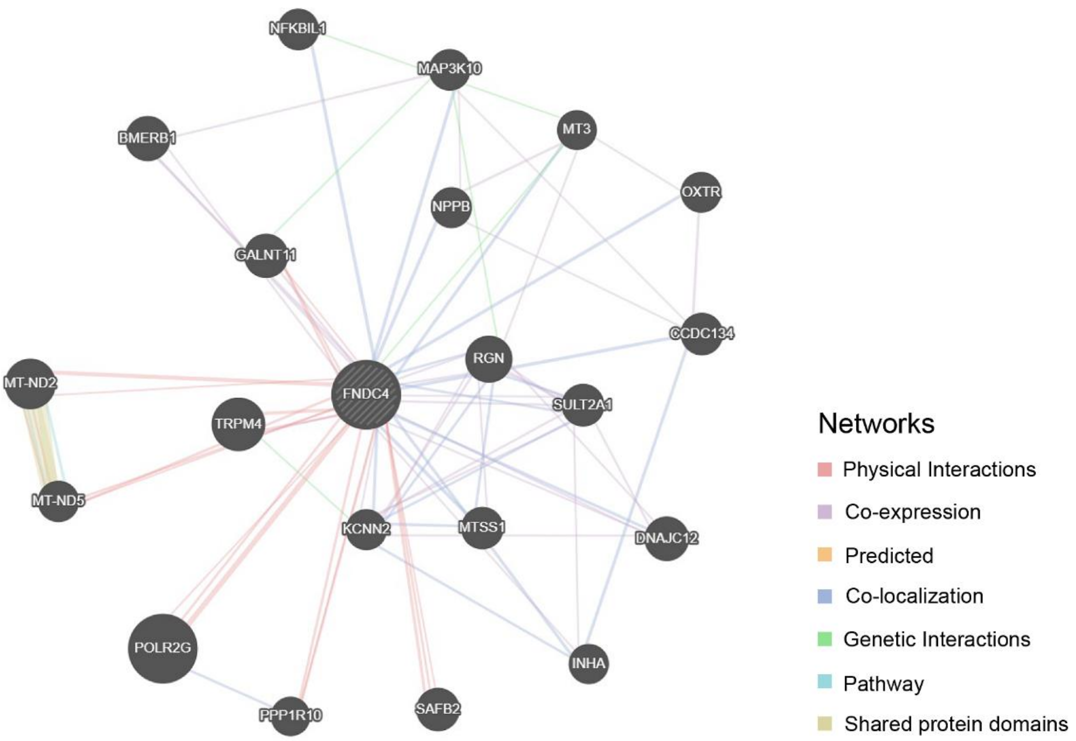

Supplementary figure 1 FNDC4 related gene interaction network. (GeneMANIA database)

From the aspects of genetic interactions, co-expression, physical interactions, co-localization, shared protein domains and pathway, the interaction network of FNDC4 related genes is established.
